# Mechanism and Development of Thermo-Rheological Properties of Asphalts Modified by Reactive Polymer Systems

**DOI:** 10.3390/ma16206631

**Published:** 2023-10-10

**Authors:** Martin Jasso, Juan Sebastian Perez Jaimes, Esteban Felipe Tellez Vega

**Affiliations:** Bituminous Materials Chair, Department of Civil Engineering, Schulich School of Engineering, University of Calgary, 2500 University Drive NW, Calgary, AB T2N 1N4, Canada

**Keywords:** reactive polymers, Elvaloy, B2Last, Superpave, thermo-rheological properties

## Abstract

The new class of reactive polymers is designed to modify asphalt through chemical reactions with asphalt components. The complexity of such systems and the long experience with thermoplastic elastomers as well as with some other “classical” modifiers, and to a degree that our present testing methods and even specifications revolve around these materials, might obscure the fact that we are dealing with rather different modification systems and possibly with new emerging asphalt paving technologies. Our present work attempted to compare two different reactive polymer systems with the “classical” system which uses thermoplastic elastomer. The impact of reactive polymer systems on asphalt was studied through material properties manifested by specification tests and through the development of thermo-rheological properties in linear and non-linear viscoelastic regions. As expected, the behavior of reactive polymeric systems with different chemistries also differed among themselves. The available results showed that the reactive groups of polymers react with polar components of asphalt leading to higher stiffness at elevated pavement temperatures and differing impact on low temperature properties. The data point to a significantly improved resistance to plastic deformation of pavement in the case of reactive polymers, despite the fact that elastic recovery-based specification tests failed to identify this improvement.

## 1. Introduction

It is well known that increased stresses (thermal, mechanical, environmental, etc.) on roads are responsible for the rapid deterioration of pavements [1,2]; thus, there is a significantly increased demand for high quality flexible pavements. Although several different measures could be implemented for the reduction in pavement distresses, for example, improved mix design [3], optimized use of materials [4], streamlining construction procedures, etc., the most effective option is the transformation of asphalt binder to material with better engineering properties [4]. This is generally carried out by modification of asphalt binder with synthetic polymers. In general, the introduction of polymers enhances resistance to plastic deformation [4,5], but also significantly affects the resistance to fatigue and low temperature cracking [4,5,6]. Furthermore, the possible thickness reduction in pavement [5,7], enhanced adhesion [5], resistance to ageing [4,5], etc., provide a significant reduction in economic and environmental costs.

Nowadays, the most commercially successful polymer for the modification of asphalt is triblock copolymer, styrene−butadiene−styrene (SBS). The structure of this thermoplastic elastomer consists of incompatible segments of elastomer and plastomer [8]. Thus, the butadiene/styrene ratio decides whether the elastomeric or plastomeric properties dominate. For asphalt modification, SBS copolymer containing approximately 30% styrene and 70% butadiene was found to be the best compromise between the weak swollen butadiene phase and its reinforcement by hard styrene segments [9]. The incorporation of SBS to asphalt is generally carried out under high shear conditions at high temperatures. The swelling ability of butadiene by light oily fractions leads to a subsequent increase in polymer volume. Depending on the chemical character of the solvent and the swelling ability of SBS, the polymer volumetrics could increase even further. This effect is of course desirable as it leads to a more effective transfer of polymer properties to the whole asphalt system [10]. The internal structure of PMA is in this case formed by swollen and independently acting dispersed polymer droplets, in other words, the polymer is acting as an active elastomeric filler [4]. The optimum thermo-rheological and engineering properties of such PMA could be achieved only at higher concentrations of modifier [11], which increases the cost of modification and the risk of gelation. Although SBS is commercially the most successful polymer modifier in the world, it is not the universal and only solution in asphalt modification. As an example, copolymers of styrene and butadiene require a specific composition of asphalt [12].

Due to only partial compatibility, the separation of SBS must also be considered [10]. To avoid these issues, the stabilization of SBS modified asphalts is commonly carried out through dynamic vulcanization [8,13]. In this process, a small amount of sulfur serves as a crosslinking agent for unsaturated butadiene in SBS modified asphalt. The uncontrolled vulcanization process leads to the formation of bridges of predominantly polysulfide character and to the formation of a three-dimensional polymer network in asphalt [14,15]. Such a major change in internal structure results in increased stability of the system and greater transfer of polymer properties to asphalt. According to [8,15], the vulcanization of SBS modified asphalt resulted in higher values of maximum service temperature and improved behavior at low service temperatures. The results from Multiple Stress Creep and Recovery experiments showed lower values of non-recovered compliance function (Jnr_3.2kPa_) and higher values of the percent recovery (%Recovery_3.2kPa_) [16]. These results point to a significant enhancement of entropy elasticity when compared to non-vulcanized PMA. Furthermore, the crosslinked SBS modified asphalt shows significant improvements in the relaxation–deformation processes [8,17,18,19], high strength and density of internal network [8,20], and complex shear viscosity behavior [8,21].

The introduction of sulfur as a crosslinking agent for SBS modified asphalts has also several drawbacks. Among the most significant, as already mentioned, is the potential gelation of SBS modified asphalt and worsened resistance to short-term thermo-oxidative ageing simulated in rolling thin film oven test (RTFOT). These effects can be partially mitigated by using alternative sulfur-based or sulfur-free crosslinking agents [8].

The alternative way of polymer modification of asphalt aims at the reaction between the polymer and some components of asphalt. The most successful reactive polymer modifier is Elvaloy [22,23], from the subgroup of reactive elastomeric terpolymers (RET). The structure of Elvaloy is based on a polyolefin main chain with large side groups containing epoxy rings and terpolymers of ethylene [24]. The modification of the main polyolefin chain improves its flexibility, reduces crystallinity, and most importantly, increases the polarity [24]. The enhanced polarity of the polymer results in higher compatibility with asphalt. Furthermore, during the blending of asphalt with Elvaloy, the activation of oxirane by heat takes place. According to [25,26], the curing of Elvaloy may be accomplished by reaction with carboxylic acids in asphalt. The reaction of activated oxirane with polar asphalt components results in the formation of new structures. Given the chemical complexity and unknown internal structure of asphalt, it is very difficult to predict the curing mechanism, the character of new structures, and therefore the performance of modified asphalt.

The formation of covalent bonds between asphalt and RET is responsible for complex changes in the colloidal system and has a direct impact on the properties of modified asphalt [24,25]. The RET modified asphalt has significantly increased values of maximum service temperatures and very low values of Jnr_3.2kPa_ [27]. The values of MSCR %Recovery_3.2kPa_, however, remain significantly lower than in the case of SBS modified asphalts [27]. Although the architecture of RET suggests low glass transition temperatures, the low temperature properties of such modified asphalts remain generally unchanged as measured by present specification tests. This phenomenon can be explained by several different theories. Firstly, the formation of covalent bonds does not only increase the stiffness of asphalt, but also increases the values of T_g_ [28]. Secondly, the maximum concentration of RET is generally limited due to the increased risk of gelation of asphalt. Lastly, compared to SBS, the swelling ability of reactive systems based on a polyolefin chain is very low [6]. When all these effects are combined, despite the low values of T_g_ of Elvaloy, the low temperature properties are not significantly affected.

With the progress of polymer science and technology, and chiefly due to ever-increasing demands for high quality pavements, a new class of reactive modifiers of asphalt binder under the commercial name B2Last has been introduced. B2Last is a low viscosity liquid based on mono- and oligomeric aromatic diisocyanate capable of reaction with polar components of asphalt [29,30]. It should be mentioned that modifiers with isocyanate group (-NCO) reactive group were investigated as possible asphalt modifiers [31]. Navarro et al. [32] studied the effects of 4,4′-diphenylmethane diisocyanate in combination with low molecular weight polyethylene glycol. Although the thermo-rheological properties of modified asphalt were improved when compared to the properties of straight run asphalt, the optimum performance was observed after 3 months of curing at ambient temperatures. The authors also noted negligible effects of additional curing on low temperature behavior.

The reaction of -NCO is expected to target amine and carboxylic functional groups, or any other chemical species with “active hydrogen” that are present in asphalt [30]. As a result, besides the formation of linear structures, there is also an increased potential for the formation of three-dimensional structures. The role of low molecular weight polyethylene glycol used in combination with isocyanate is to reduce the amount of reactive -NCO group [33]. Such prepolymer with controlled concentration of reactive -NCO groups is safer to use [33], however, it may have a reduced impact on the properties of modified asphalts. This could suggest that the formation of a stronger and more complex network was formed by the B2Last modifier. According to [29], the produced internal structure has a flexible character, however, it is not clear whether aromatic diisocyanate forms covalent bonds with asphalt and potentially polymerize. In -NCO type modifiers and without the presence of low molecular weight polyethylene glycol, the safety concerns due to the possibility of the evolution of harmful emissions should be also considered [34]. These, however, should be minimized by optimization of the blending time and temperature with asphalt.

Two different blending procedures of asphalt with B2Last were studied by [29]. The first method was a batch process, a typical modification of asphalt with additive. The second process was in-line blending, in which B2last was injected directly to asphalt before the production of paving mixes. Authors showed that both modification methods were effective, however, the optimum concentration of -NCO type of modifiers should be evaluated through trial and error for particular straight run asphalt.

Our present work attempts to compare the impact of two reactive polymer systems with different chemistries, one polymeric and one in the form of mono- and oligomer. The two reactive systems were compared to the technology using SBS elastomeric copolymer. The impact of modification technologies on asphalt was studied through material properties manifested by specification tests and through the development of thermo-rheological properties in linear and non-linear viscoelastic regions. The results showed a significant difference between the already well-known behavior of asphalt modified by thermoplastic elastomers and the changes in the behavior of asphalt modified by different reactive systems.

## 2. Materials and Methods

### 2.1. Preparation of Polymer Modified Asphalts

A conventional commercial asphalt prepared by vacuum distillation of crude oil to 200/300 Pen grade was selected as a base. Its properties according to Superpave specification are shown in Table 1. Asphalt manufactured by Cenovus Energy (Calgary, AB, Canada) was modified by SBS copolymer with and without crosslinking with sulfur and by technologies using reactive polymers, namely, Elvaloy and B2Last. The first technology was using “classical” modification via thermoplastic elastomer, styrene−butadiene−styrene Kraton D1101 from Kraton Corporation (Houston, TX, USA). This polymer with approximately 31 wt.% of styrene was identified as a linear block copolymer with medium molecular weight. Technical sulfur, (solubility in carbon disulfide (CS_2_) ranging from 95.5 to 96.5%), was used as a crosslinking agent.

The first reactive polymer, Elvaloy 4170, was an ethylene−butylacrylate−glycidylmethacrylate terpolymer with a butylacrylate content of 20 wt.% and glycidylmethacrylate content of 9 wt.%. This polymer is manufactured by DOW Chemical Co. (Midland, MI, USA). In the case of Elvaloy, polyphosphoric acid PPA 115 containing 83.3% phosphorus pentoxide (P_2_O_5_) or otherwise stated as 115% H_3_PO_4_ (phosphoric acid) equivalent from Innophos Inc. (Cranbury, NJ, USA), was also used. The second reactive system for preparation of modified asphalts was B2Last from BASF Corporation (Wyandotte, ML, USA), a low viscosity liquid containing -N=C=O reactive group. This modifier of asphalt was used without any recommended supporting technology.

Several blending protocols were used for preparation of modified asphalts, however, in all cases, asphalt was heated and maintained below 200 °C to avoid thermal degradation of asphalt and polymer:

- The SBS copolymer was dispersed by an interchangeable rotor-stator high-shear mixer operating in the interval from 1000 to 3000 rpm until no separate parts of the polymer were observed. The dynamic vulcanization was performed as the second blending step. It should be noted that no additional crosslinking additives were used.
- Elvaloy was incorporated into asphalt by a low-shear mixer operating in the interval from 500 to 2000 rpm. The blending time to achieve adequate formation of covalent bonds between asphalt and polymer ranged from 3 to 8 h. A small amount of PPA was added to asphalt during the last hours of blending.
- The modification of B2Last was again achieved by a low-shear mixer operating in the interval from 500 to 2000 rpm. The blending started at low rpm and gradually increased with the increasing reaction time. The conversion of -NCO was regularly monitored through Fourier Transform Infrared Spectroscopy and measurements of rutting parameter $|G^{*}|/\sin\delta$. The optimum reaction time ranged from 3 to 6 h.

The composition of prepared modified asphalts can be seen in Table 2.

### 2.2. Testing of Prepared Polymer Modified Asphalts

#### 2.2.1. Superpave Binder Specification Testing

Produced polymer modified asphalts were tested according to AASHTO M320 [35] and AASHTO M332 [36] and were arranged according to Superpave performance grades, PG XX-YY, indicating the maximum and minimum climatic temperatures that asphalt can be exposed to.

#### 2.2.2. Thermo-Rheological Testing

##### Preparation of Samples

The same procedure was used for each rheological testing. Firstly, the tested sample of PMA was slowly heated to 160 °C for 15 min and stirred before being directly poured on the testing geometry. The time interval to achieve temperature equilibrium before testing was 30 min. In the case of rectangular torsion bars, heated PMAs were poured into custom molds, cooled down at ambient temperature for one hour, and then stored in a freezer before testing. Samples were processed within 24 h. A new sample was always used for a particular rheological measurement.

##### Material Spectroscopy

The frequency sweeps were conducted in a strain controlled rotational rheometer Ares-G2 from TA Instruments. Since the temperature has a significant impact on the properties of asphalt, different geometries were used for measurement at different temperatures. Rectangular torsion bars with dimensions of 25.5 ± 0.1 × 12 × 2.65 mm were used for testing temperatures below zero, 10 mm diameter plate–plate geometries were used for temperature interval from 10 to 20 °C, 25 mm diameter plate–plate geometries were used for temperature interval from 30 to 60 °C, and 50 mm diameter plate–plate geometries were used for testing temperatures above 60 °C.

The angular frequency in all tests ranged either from 0.01 rad/s or from 0.1 rad/s to 100 rad/s. The applied deformation was determined through amplitude sweep testing as 80% of maximum deformation within the linear viscoelastic region. The optimum magnitude of deformation was determined for each testing temperature at an angular frequency of 10 rad/s.

##### Steady Shear Viscosity

Strain-controlled rotational rheometer Ares G2 was used for the measurement of steady shear viscosity at two selected testing temperatures of 50 °C and 60 °C. The shear rate in all shear viscosity measurements ranged from 10^−4^ s^−1^ to 100 s^−1^. All viscosity measurements were performed using 25 mm diameter plate–plate geometries with a gap of 1 mm.

##### Start-Up of Steady Shear Experiment

A start-up of a steady shear experiment in a strain-controlled rotational rheometer Ares G2 was performed. The tested temperature for all start-up measurements was 60 °C using 25 mm plate–plate geometry and 1 mm gap. The shear rates in the start-up of the steady shear experiment were 0.5 s^−1^, 2 s^−1^, and 5 s^−1^.

## 3. Results and Discussion

### 3.1. Superpave Binder Specification

**Figure 1:**
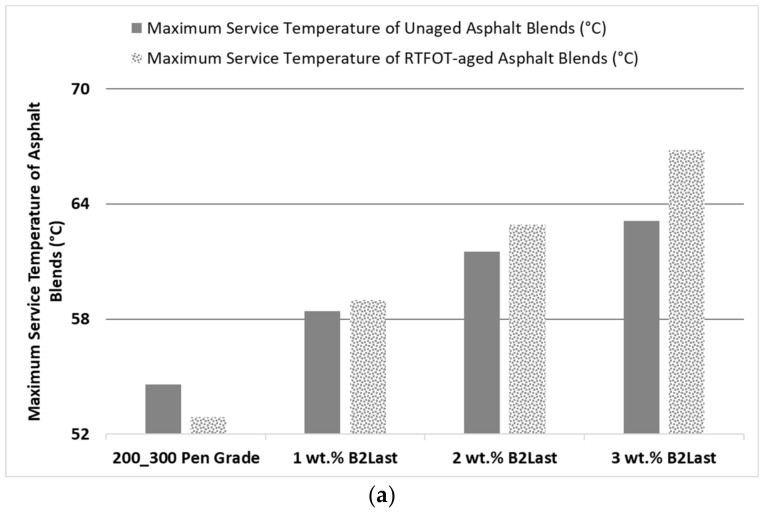

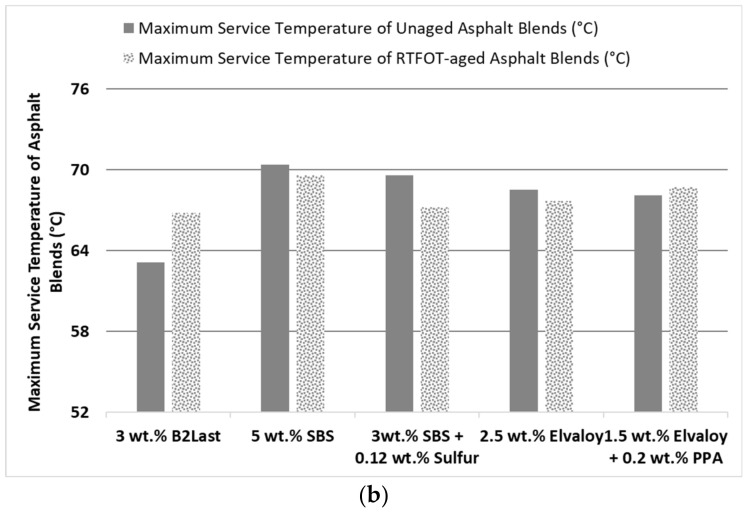
Effects of B2Last (**a**) and comparison of the effects of different modification technologies (**b**) on maximum service temperatures of prepared asphalt blends.

The effect of increasing concentration of B2Last on maximum service temperature of unaged (defined as |G*|/sinδ ≥ 1 kPa, [35]) as well as RTFOT aged (defined as |G*|/sinδ ≥ 2.2 kPa [35]) asphalt blends is shown in Figure 1a. It is clear that linearly increasing concentration of B2Last resulted in linearly increasing maximum service temperature of unaged modified asphalts. Particularly interesting was to observe the further linear increase in maximum service temperature after RTFOT ageing. The reaction between the isocyanate group of mono- and oligomeric methylene diphenyl diisocyanate of B2Last and polar components of asphalt was optimized to achieve the highest possible conversion prior to the preparation of B2Last modified asphalt. This was carried out by monitoring the rheological properties and FTIR characteristics during blending. It seems, however, that the aggressive effect of air and high temperatures during RTFOT ageing resulted in additional B2Last conversion and/or reorganization of already created internal structure.

The highest resistance to rutting, defined by high temperature parameter |G*|/sinδ, was obtained at 3 wt.% of B2Last and was selected for comparison with modification technology using thermoplastic elastomer, styrene−butadiene−styrene, with and without sulfur as a crosslinking agent, and Elvaloy 4170 with and without polyphosphoric acid. For comparison and evaluation purposes of modification technologies, it was decided to prepare modified asphalts with high temperature performance grades of PG 68 ± 2 °C as determined after RTFOT ageing. This can be seen in Figure 1b. The concentration of modifiers was carefully considered, so that each modifier had sufficient concentration in asphalt to demonstrate the main features of particular modification technology.

**Figure 2:**
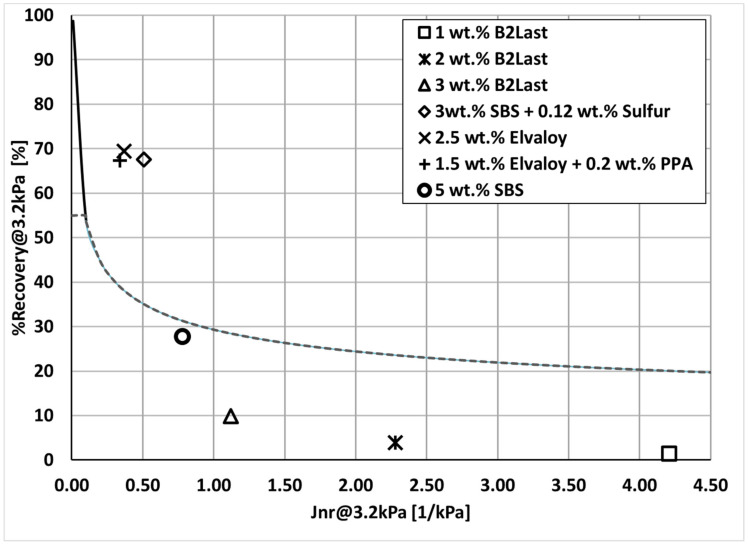
%Recovery_3.2kPa_ vs. Jnr_3.2kPa_ at 58 °C of prepared asphalt blends.

It was observed that the performance of asphalt with the same PG at high service temperature was not similar. On the contrary, the clear distinction of performance in MSCR Jnr_3.2kPa_ and %Recovery_3.2kPa_ measured at 58 °C was seen for each modification technology as shown in Figure 2.

For reference, the values of Jnr_3.2kPa_ and %Recovery_3.2kPa_ measured at 58 °C for 200/300 Pen grade straight run asphalt, used as a base for modification, were 7.4 kPa^−1^ and 1%, respectively. These values were expected as this straight run asphalt with a relatively high value of penetration (low value of viscosity) was characterized only as PG 52–34. Although the introduction of 1, 2, and 3 wt.% of B2Last improved values of Jnr_3.2kPa_ substantially, the values of %Recovery_3.2kPa_ remained very low. Asphalt blend containing 5 wt.% of SBS had values of Jnr_3.2kPa_ equal to 0.78 kPa^−1^ and surprisingly low value of %Recovery_3.2kPa_ equal to 27.7%. Modified asphalts with the lowest values of Jnr_3.2kPa_ (below 0.5 kPa^−1^) and the highest values %Recovery_3.2kPa_ (above 65%) were prepared by technologies containing crosslinked SBS and Elvaloy with and without PPA.

**Figure 3:**
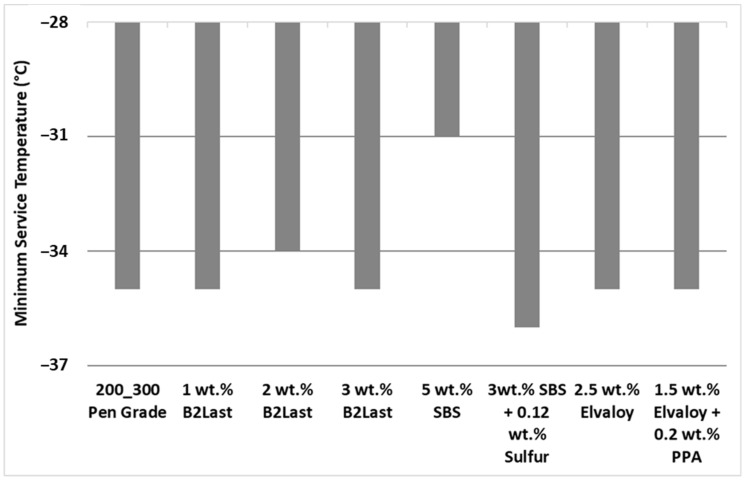
Minimum service temperatures obtained by BBR testing of prepared asphalt blends.

The minimum service temperature determined by a bending beam rheometer (BBR) [35] on PAV aged samples of modified asphalts can be seen in Figure 3. The effect of B2Last on minimum service temperature is somewhat controversial. Materials with 1 wt.% and 3 wt.% of B2Last did not have any effect on minimum service temperature. Sample of modified asphalt containing 2 wt. % of B2Last worsened the minimum service temperature by 1 °C.

The effects of the second reactive modifier, Elvaloy, either alone or in combination with PPA, did not have any effect on minimum service temperature. While 5 wt.% of SBS worsened the minimum service temperature by 4 °C, the introduction of sulfur as a crosslinking agent in asphalt modified by 3 wt.% of SBS improved the minimum service temperature by 1 °C.

Ho et al. [37,38] found that the BBR test has difficulties in recognizing the true impact of modifiers on low temperature properties. The low service temperatures of modified asphalts determined by BBR were on average 3 to 6 °C higher than those determined by DTT. This was explained by increasing values of failure stress, energy, and strain and lower values of secant modulus in polymer modified asphalts. Due to nonconclusive BBR results and due to the observation that reactive B2Last modifier is improving the stiffness of asphalt binder, the DTT was also used to assess the low temperature behavior. As a rule of thumb, the value of critical cracking temperature (determined as a crossover between the master curve of BBR stiffness and DTT thermal strength) worsened by 0.4 °C per addition of 1 wt.% of B2Last modifier (the results are not shown in this contribution).

**Figure 4:**
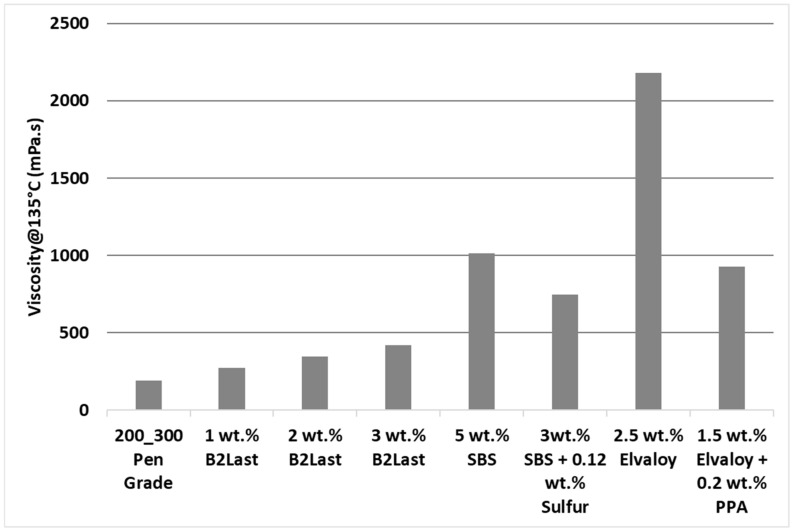
Viscosity at 135 °C of asphalt blends prepared by different modification technologies.

Figure 4 shows the viscosity of asphalts modified by different technologies measured at 135 °C [35]. As can be seen, linearly increasing concentration of B2Last leads to linearly increasing values of viscosity. While the value of viscosity at 135 °C for 3 wt.% B2Last was below 500 mPa·s, the viscosity of asphalt blend containing 3 wt.% SBS crosslinked with 0.12 wt.% sulfur and blend containing 5 wt.% SBS without any crosslinking agent were 77% and 140% higher, respectively. The highest values of viscosity were recorded for modification technology using the reactive system Elvaloy. Although the viscosity value of the blend containing 1.5 wt.% of Elvaloy and 0.2 wt.% of PPA had similar viscosity to SBS modification technology, a sample containing 2.5 wt.% of Elvaloy without PPA had a viscosity of 2181 mPa·s. This result was expected due to the development of complex asphaltene–Elvaloy structures [6,39,40,41].

Lower values of viscosity at 135 °C are preferred from handling and transportation perspective. The significant increase in the maximum service temperature after RTFOT ageing, however, points to potentially higher values of viscosity of B2Last modified asphalts during the mixing and construction phase.

### 3.2. Mechanical Spectroscopy

Mechanical spectroscopy, together with creep and relaxation experiments, belong to the most common experimental techniques in rheology for a description of viscoelastic materials. While constant shear stress is applied in the creep experiment, changes in stress at constant deformation are monitored in the relaxation experiment. In mechanical spectroscopy, also known as dynamic material analysis, stress or strain varies sinusoidally with a given frequency.

In order to assess the behavior of viscoelastic material, the relaxation modulus over a wide range of time, ideally from time zero to infinity, should be achieved. Although this is experimentally not possible through the application of the time–temperature superposition principle, it is possible to extend the time interval for viscoelastic observation. When the studied material fulfills the condition of rheological simplicity, the experimental data can be shifted to master curves of dynamic material functions, described by discrete or continuous relaxation spectrum, {λ_i_, g_i_}, according to the following relations [42]:

(1) $$G^{\prime }=\sum_{i}\frac{G_{i}\omega^{2}\lambda_{i}^{2}}{\left(1+\omega^{2}\lambda_{i}^{2}\right)},G^{\prime \prime }=\sum_{i}\frac{G_{i}\omega \lambda_{i}}{\left(1+\omega^{2}\lambda_{i}^{2}\right)}$$

where i = 1,2, …, N;

ω is the frequency in rad/s;
$G_{i}$ are the spectral strengths;
$\lambda_{i}$ are the relaxation times.

The selection of reference temperature has to be carefully considered. The change in reference temperature alters relaxation times, thus resulting in the requirement for horizontal shifting factors, generally described by the Williams–Landel–Ferry (WLF) equation [42]:

(2) $$\log\left(a_{T}\right)=\frac{-A(T-T_{0})}{B+(T-T_{0})}$$

where T is the temperature;

T_0_ is the reference temperature;
A and B are constants.

The master curves of dynamic material functions were constructed using the commercial software IRIS RheoHub [43].

**Figure 5:**
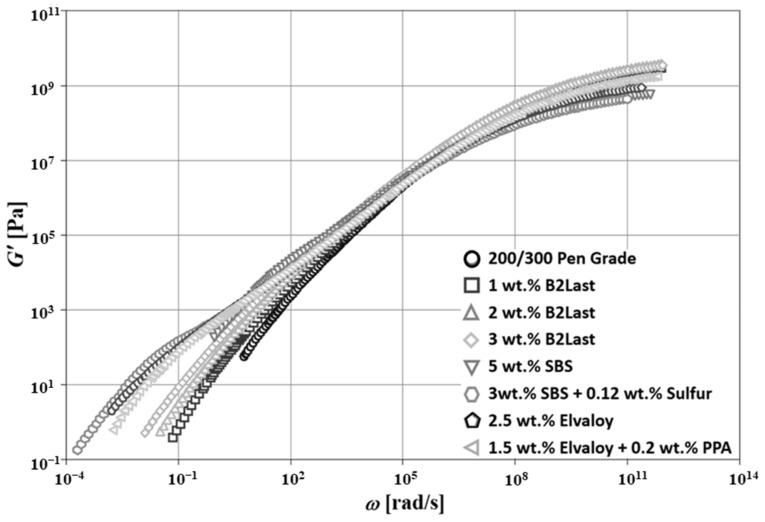
Master curves of $G^{\prime }(\omega )$ for asphalt blends prepared by different modification technologies, Tr = 50 °C.

**Figure 6:**
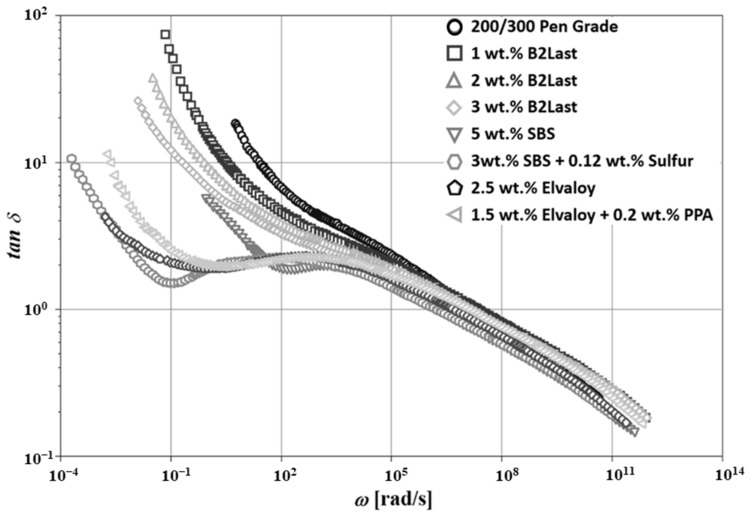
Master curves of tanδ(ω) for asphalt blends prepared by different modification technologies, Tr = 50 °C.

Figure 5 shows the master curves of $G^{\prime }(\omega )$ for asphalt blends prepared by different modification technologies at Tr = 50 °C. Three different regions generally dominate in straight run asphalts. In the first region, a solid-like behavior is observed at low temperatures (high frequencies). The magnitude of $G^{\prime }(\omega )$ dominates in this region and its value approaches 10^9^ Pa. Magnitude of $G^{\prime \prime }(\omega )$ reaches the maximum before its values start dropping. The second important region, characterized by the crossover of $G^{\prime }(\omega )$ and $G^{\prime \prime }\left(\omega \right),$ is generally observed at intermediate frequencies. Newtonian zone observed at high temperatures (low frequencies) is the third region. Contrary to solid-like behavior, in the Newtonian zone, the magnitude of $G^{\prime \prime }(\omega )$ is generally higher than the magnitude of $G^{\prime }(\omega )$. The master curve of tanδ(ω) for straight run asphalt, shown in Figure 6, also confirms this behavior.

The microstructure of straight run asphalt can be indirectly observed from the master curves of dynamic material functions. Based on only two distinctive regions on the master curve of tanδ(ω), it could be deduced that the internal structure of this asphalt is relatively weak and can be associated only with intermolecular interactions [28,43]. The two distinctive regions on the master curve of tanδ(ω) suggest the low mobility of asphalt components at low temperatures, however, the reduction in viscosity at higher temperatures, together with the weak internal structure of this sol-like material, result in a sharp linear increase in tanδ(ω) [44]. It is clear that this particular effect is the main reason for the formation of one of the most common pavement distresses, which is rutting.

Figure 5 and Figure 6 show the effects of a variety of modifiers on the master curves of $G^{\prime }(\omega )$ and tanδ(ω), respectively. The modification of straight run asphalt with B2Last reactive modifier led to a higher magnitude of $G^{\prime }(\omega )$. The increase in $G^{\prime }(\omega )$ was proportional to the concentration of this modifier. Quite interesting was the observation of the shape of $G^{\prime }(\omega )$ master curves for asphalts modified by B2Last. Although the modification effect of B2Last increased stiffness, potentially due to the chemical reaction of the -NCO functional group with polar components of asphalt, the internal structure did not seem to be significantly affected. The same behavior was also observed on the master curves of tanδ(ω). Although the modification effect of 1, 2, and 3 wt.% of B2Last seemed to be relatively small, as reflected by dynamic material functions, the further development of properties of these modified asphalts was observed after RTFOT ageing.

The modification effects of the second reactive system, Elvaloy with and without the presence of PPA, showed substantial changes in the master curves of $G^{\prime }(\omega )$ and tanδ(ω). The asphalt blend modified with 2.5 wt.% of Elvaloy 4170 showed the onset of the shoulder on the master curve of $G^{\prime }(\omega )$, which is characteristic for the formation of the network-like internal structure [6,25]. The mechanism of modification of Elvaloy appears to be similar to B2Last reactive modifier, although both modifiers carry different reactive chemical groups. While the reactive group for B2Last is -NCO, the chemical reaction of Elvaloy is based on the opening of the epoxy functional group and subsequent reaction with polar components of asphalt.

The main difference between these two reactive systems, besides different functional groups, is the chemical character of modifiers. While B2Last is a low molecular weight mono- and oligomer which reacts and/or converges to macromolecules when blended with asphalt, Elvaloy is already in the form of polymer which must be dispersed in asphalt matrix for the reaction to occur. The higher molecular weight of the modifier, together with the reaction with asphalt components, lead to the formation of asphaltene–Elvaloy complexes [6,25]. The formation of these complexes depends on the chemical composition of asphalt and the concentration of Elvaloy, i.e., the number of functional groups present on the chain. As a result, even the small addition of Elvaloy, generally above 2.5 wt.%, could lead to gelation, and thus formation of an infusible and insoluble material [22]. The dynamic material functions for asphalt modified by Elvaloy could suggest not only the formation of asphaltene–Elvaloy complexes, but also the significant transition of colloidal structure from sol-like to partial gel-like.

Interestingly, despite a lower content of the polymer, the asphalt modified by 1.5 wt.% of Elvaloy and 0.2 wt.% of PPA showed similar behavior to asphalt modified by 2.5 wt.% of Elvaloy. The slight deviation between these two modified asphalts was observed at $\omega \approx 10^{-1}rad/s$, with a higher tendency to flow for asphalt modified with 1.5 wt.% of Elvaloy and 0.2 wt.% of PPA. This effect could be attributed to several potential mechanisms: (1) the catalytic effect of PPA on the Elvaloy reaction with asphalt’s polar components [45]; (2) the precipitation of asphaltenes [46] and formation of new asphaltenes with PPA through the conversion of the portion of naphthene to polar aromatics, and polar aromatics to asphaltenes [47]. This mechanism could not only result in the creation of new active centers able to react with oxirane, but also in the changes in the colloidal system. According to [25], the combination of both mechanisms could be considered. In general, however, the development of properties does not depend only on the chemistry of reactive modification systems, but also on the source and manufacturing technology of asphalt binder.

The evolution of the fourth region, demonstrated by a shoulder of $G^{\prime }(\omega )$ as well as shorter region of local maximum followed by local minimum observed on the master curve of tanδ(ω) (Figure 5 and Figure 6, respectively) was also observed in modification technology using 5 wt.% of thermoplastic elastomer SBS. Although very similar features between Elvaloy and SBS copolymer could be observed on the master curves of dynamic material functions, their origin is completely different. The presence of oxirane in Elvaloy promotes a reaction with polar asphalt components, which enhances the stiffness of modified asphalt [48,49,50]. The formation of asphaltene–Elvaloy complexes depends on the amount of functional groups present in asphalt as well as Elvaloy.

The non-vulcanized SBS copolymer is generally dispersed in asphalt in the form of ultra-fine particles. The butadiene part of the polymer is able to absorb light oily asphalt’s components which leads to volumetric expansion. The swelling ability of the polybutadiene phase is a function of several different factors, e.g., molecular weight of polymer, chemistry and amount of light oily fraction in asphalt, morphology of polymer, temperature, etc. Consequently, dispersed polymer particles swell four to ten times. At a critical concentration of SBS, the polymer-rich phase becomes continuous, which explains the change in deformation–relaxation processes at intermediate to high temperatures, and thus a decrease in temperature susceptibility of modified asphalt [10].

Without any stabilization mechanisms, such as vulcanization, the SBS particles act independently as an active, swollen, elastomeric filler and the transfer of its properties to asphalt binder is limited [8]. Furthermore, over longer storage at high temperatures, the SBS particles coalesce and the polymer phase may separate from asphalt.

The introduction of a small amount of sulfur is able to eliminate the issues with the separation of SBS polymer. As the crosslinking with sulfur leads to the formation of a three-dimensional polymer network, the transfer of the elastomeric properties to modified asphalt is also more effective [8]. This effect can be seen on the well pronounced shoulder of $G^{\prime }(\omega )$ as well as the large region of local maximum followed by local minimum observed on the master curve of $tan\delta \left(\omega \right),$ which is generally associated with the behavior of diluted polymer systems. These phenomena can be seen in Figure 5 and Figure 6, respectively.

**Figure 7:**
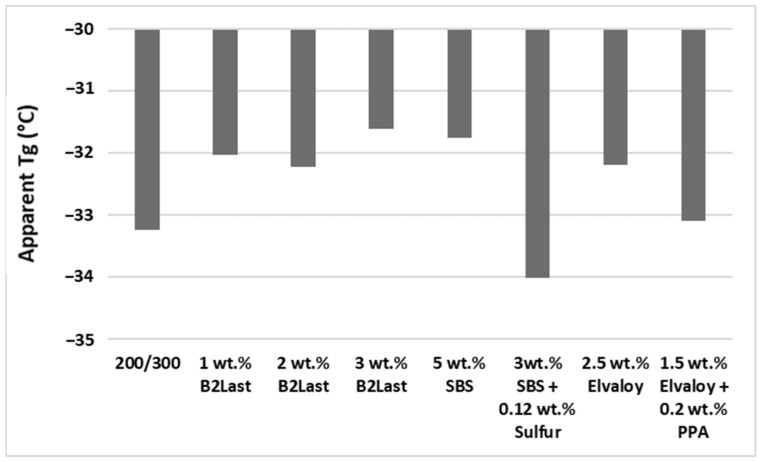
Values of apparent T_g_ obtained from the master curves of $G^{\prime \prime }(\omega )$ for asphalt blends prepared by different modification technologies.

Figure 7 shows the values of apparent T_g_ for asphalt blends prepared by different modification technologies. In order to obtain values of apparent T_g_, new master curves of dynamic material functions superposed to a reference temperature of −20 °C were prepared. The transformation of frequency to temperature domain was based on the WLF equation. As discussed in [8,25], the value of T_g_ depends on the selected value of testing frequency $\omega_{0}$ used for the transformation of frequency to temperature domain. Although the values of apparent T_g_, defined as a maximum on the master curves of $G^{\prime \prime }(\omega )$, have only informative character, they can be used to evaluate and compare the effects of particular modification systems at low temperatures.

It can be seen from Figure 7 that asphalt modified by Elvaloy and asphalts modified by B2Last had higher values of apparent T_g_ than reference straight run asphalt 200/300 Pen grade. The higher (worse) values of apparent T_g_ of both reactive modifiers are very peculiar. Since B2Last prior to the modification is in the form of low molecular weight liquid, it should decrease the value of apparent T_g_. On the other hand, although it was expected that Elvaloy would slightly decrease values of T_g_, given the very low swelling ability in combination with the very low concentration of this modifier, the T_g_ was not significantly improved. As was already presented, even though the chemistry of both reactive modifiers is different, the mechanism of modification could be considered similar, i.e., the reaction of modifiers’ active groups with polar components of asphalt. Particularly, the formation of covalent bonds and increase in molecular weight could be the main factors for higher values of T_g_ [28]. In the case of a low concentration of PPA in combination with Elvaloy, it could be hypothesized that disintegration and to a small extent formation of new asphaltenes could reduce the molecular weight of formed asphaltene–Elvaloy complexes. Thus, slightly improved values of T_g_ could be observed.

The values of apparent T_g_ of asphalt modified by 5 wt.% SBS, together with a sample of asphalt modified by 3 wt.% B2Last, were the highest (worst) of all tested modified asphalts. The ultra-fine dispersed and swollen particles of SBS can form only a weak network of a physical character. Despite the T_g_ of the butadiene phase below −80 °C, the T_g_ of such modified asphalt is mostly controlled by the reduction in its free volume [18,21]. As was observed from Figure 5 and Figure 6, the saturation of C=C double bonds present in butadiene by sulfur leads to the formation of a polymer network. Although it seems that the formation of a three-dimensional SBS network is solely responsible for the decrease in the T_g_, other important effects must be also considered [25,28]. The formation of a three-dimensional SBS network is generally achieved by the process of dynamic vulcanization at high temperatures. As the vulcanization is not controlled, the formation of polysulfide bonds can be expected. Generally, the vulcanization increases T_g_, however, given the low concentration of sulfur in this process, the created network could be considered to have only weak character. Thus, even when vulcanization reduces the molecular motion, the transfer of properties of the crosslinked polybutadiene network has a more significant effect.

### 3.3. Steady Shear Viscosity

The steady shear viscosity measurements were selected as a complementary method to dynamic material analysis. While in the latter the structural response is given by disturbance of the studied material to a negligible extent via a small amplitude oscillation in the linear viscoelastic region, the viscosity measurements could be used to investigate the response of internal structure when subjected to flow and to determine the value of critical shear rate at which the studied material stops behaving as a Newtonian liquid.

**Figure 8:**
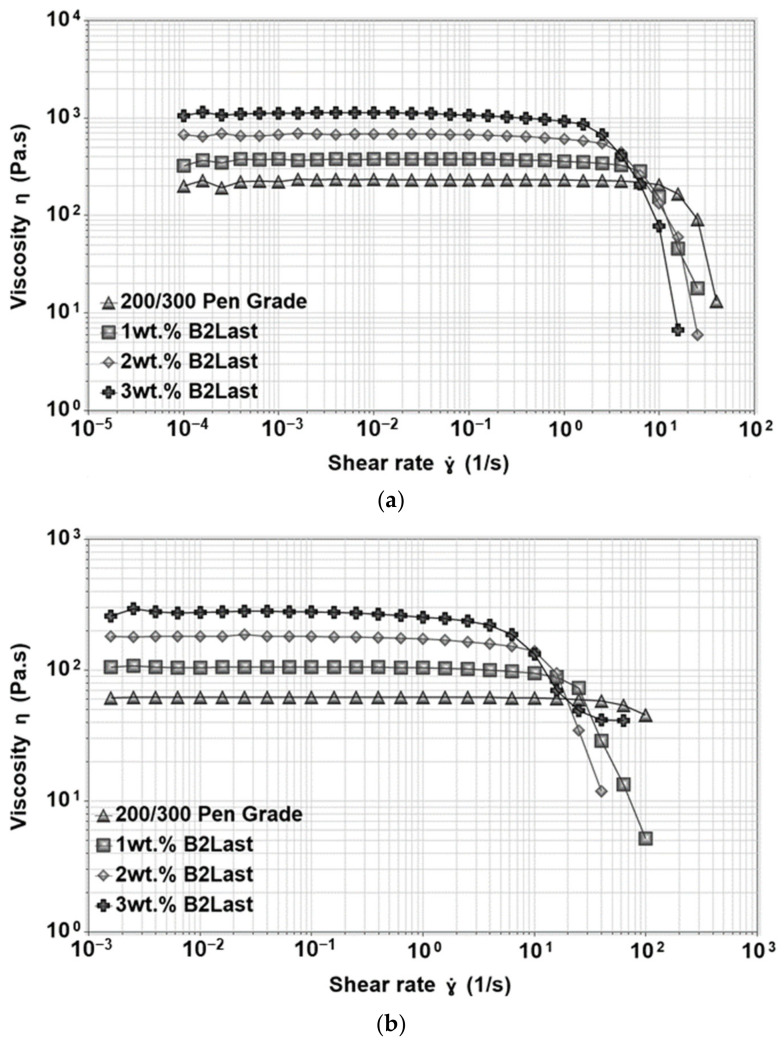
Viscosity functions of asphalts modified by different concentrations of B2Last: (**a**) 50 °C, (**b**) 60 °C.

The viscosity functions of straight run asphalt measured at 50 °C and 60 °C can be seen in Figure 8a,b, respectively. As can be seen, the viscosity function of straight run asphalt at 50 °C displayed long interval at which the viscosity was independent of the applied shear rate. This region, characterized by one value of viscosity, is generally called zero shear viscosity or Newtonian region. When the testing temperature increased to 60 °C, the magnitude of viscosity of straight run asphalt was lower, and the zero shear viscosity region was observed almost through the whole interval of applied shear rate. This behavior of straight run asphalt was expected not only due to the very weak internal structure, but also due to the significant influence of temperature on the viscosity function [22].

The viscosity functions at 50 °C and 60 °C were also investigated for asphalt modified by 1, 2, and 3 wt.% of B2Last. Results can be seen in Figure 8. Similarly to previous results, the magnitude of the viscosity function was linearly increasing with the increasing content of the B2Last modifier. Since the behavior of B2Last modified asphalts is very similar to straight run asphalt, the potential changes in its behavior were identified by fitting the viscosity curves to the Carreau–Yasuda model [51]. As expected, besides the reduction in the value of the critical shear rate at which the shear thinning occurred, no changes that could be related to the change in internal structure were found for asphalt modified by 1 wt.% B2Last. Two shear thinning regions were observed for asphalt modified with 2 wt.% of B2Last. The first, mild shear thinning occurred at a shear rate of 0.25 s^−1^ and the second sharp shear thinning region was recorded at 2.5 s^−1^. Very weak viscosity overshoot (shear thickening region) was observed at a shear rate of 0.0016 s^−1^ for asphalt modified by 3 wt.% B2Last. The correlation between the viscosity overshoot and the development of a network (physical or chemical) in modified asphalt has been found in our previous research. The presence of viscosity overshoot thus confirms the formation of a network based on the reaction of B2Last with asphalt components, however, given to a small magnitude of the overshoot, the internal network within asphalt is very weak. It should be noted, however, that the optimum conversion of B2Last was observed after the RTFOT ageing, which suggests that the internal structure is more pronounced.

The magnitude of viscosity in asphalt modified by Elvaloy with and without the presence of PPA was three times higher than asphalt modified by the B2Last modifier. The shape of the viscosity function for both reactive systems had very similar features—a short zero shear viscosity region followed by two shear thinning regions. Functional groups of Elvaloy are responsible for the formation of covalent bonds with polar asphalt components. The resulting Elvaloy–asphaltene complexes can vary in size, mobility, and different levels of internal strength producing two shear thinning regions [25]. A minor shear thickening was observed in asphalt modified by Elvaloy and PPA. Interestingly, replacing polymer with a small amount of PPA delayed the critical shear rate at which the shear thinning occurred even though materials had the same PG grade.

**Figure 9:**
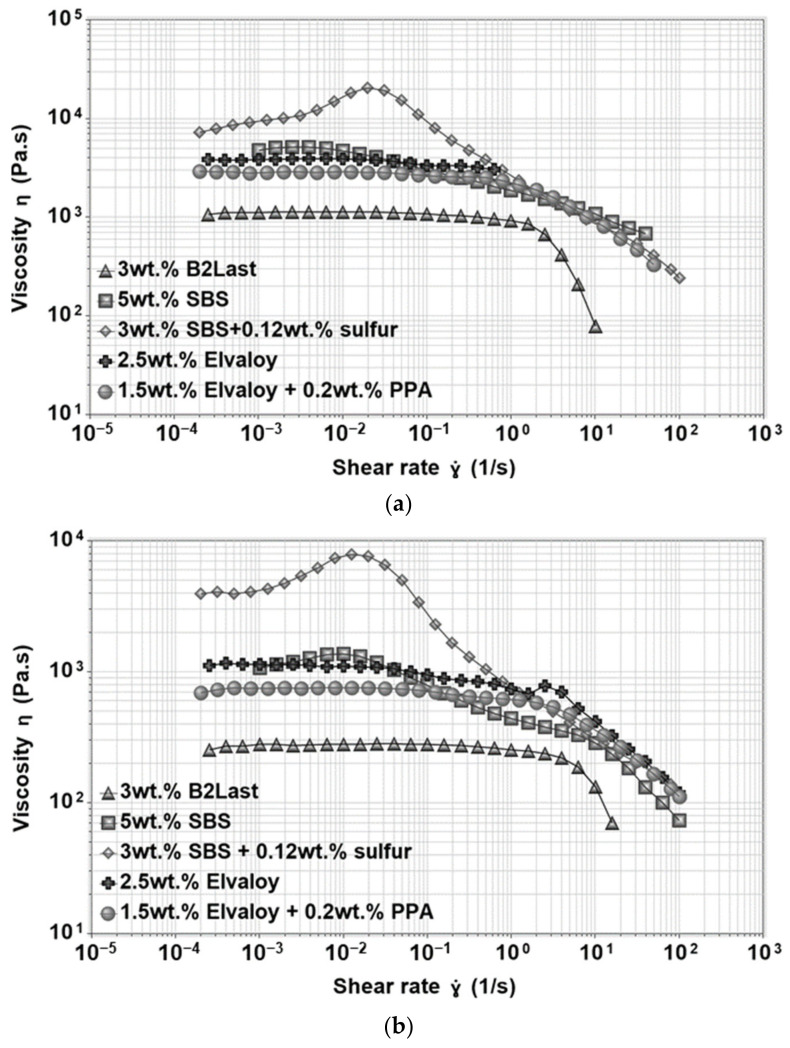
Viscosity functions for asphalts modified by different modification technologies: (**a**) 50 °C, (**b**) 60 °C.

The shear viscosity of asphalt modified by thermoplastic elastomer, SBS, with and without the presence of sulfur is also shown in Figure 9. It is clear that SBS modified asphalts had generally higher values of viscosity at 50 and 60 °C. In the case of asphalt modified by 5 wt.% of SBS, the short zero shear viscosity region was followed by a large viscosity overshoot. The shear thickening could be explained not only by the orientation of the independently acting swollen particles of polybutadiene in the direction of flow, but also by forming a weak non-permanent polymer network through physical interactions. The latter could be responsible for the reorganization of the internal network through an increase in a number of physical crosslinks. The shear thinning observed at high shear rates could be explained as the breaking of interactions within the internal structure [22]. The formation of a three-dimensional polymer network by crosslinking of SBS with a small amount of sulfur resulted in the highest value of zero shear viscosity followed by a large viscosity overshoot. As it was mentioned above, the formation of viscosity overshoot can be associated with the presence of a network in modified asphalts [8,25].

### 3.4. Start-Up of Steady Shear Experiments

It is generally accepted that the majority of pavement distresses occur out of the linear viscoelastic zone. Thus, the strength of internal networks of asphalts modified by different modification technologies was investigated by the start-up of steady shear experiments. In these types of transient experiments, the shear rate is immediately applied to the tested material and held constant during the test. Rheologically, the behavior of paving asphalts could be presented as a diluted system of entanglements. Further modification of already complex internal structure with polymers or additives could result in the appearance of stress overshoot [20,52]. The stress overshoot does not only provide information about the strength of the internal network subjected to sudden large deformation, but also about the structural development of modified asphalts [20].

The results from the steady shear viscosity measurements demonstrated that modification of asphalt binder by different modification systems could significantly affect its resistance to flow as well as change the shape of flow curves. The presence of shear thickening or two different shear thinning regions suggests the transition of these materials to non-Newtonian liquids. The complex responses of the formed internal structures cannot be described by either classical viscosity models or mesoscopic non-Newtonian viscosity models.

**Figure 10:**
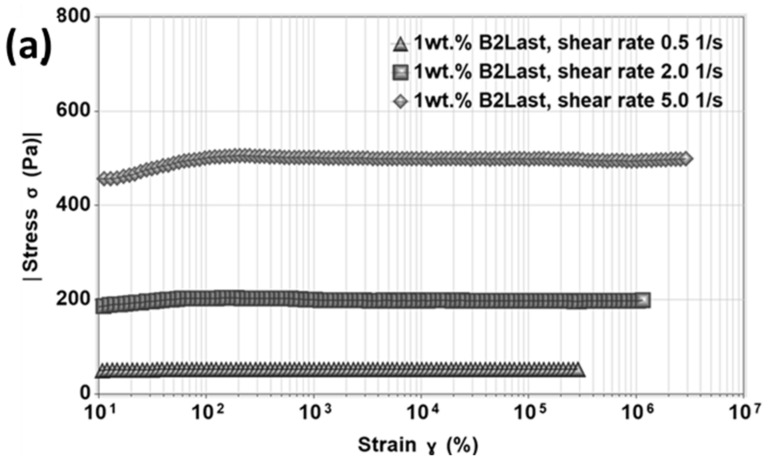

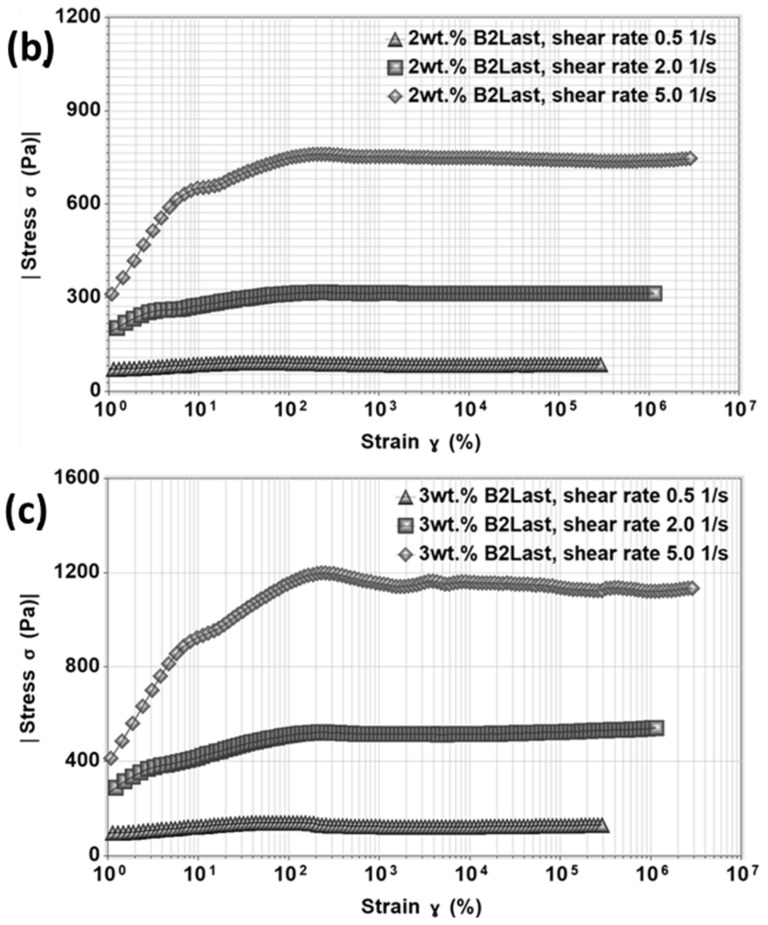
Stress overshoot as a function of strain for B2Last modified asphalts, T = 60 °C: (**a**) $\dot{\gamma }=0.5\;s^{-1}$, (**b**) $\dot{\gamma }=2.0\;s^{-1}$, and (**c**) $\dot{\gamma }=5.0\;s^{-1}$.

The stress growth function, $\tau^{+}$, of asphalts modified by different concentrations of B2Last is shown in Figure 10. In order to avoid issues with overloading of the rheometer, the start-up of the steady shear experiment was carried out at 60 °C at three different shear rates of 0.5 s^−1^, 2.0 s^−1^, and 5.0 s^−1^. In the start-up of the steady shear experiment, the response of asphalt modified by 1 wt.% of B2Last was within the linear viscoelastic domain. Such behaviors can be expressed as ${\frac{\tau^{+}}{\dot{\gamma }}\equiv \eta }^{+}\left(t,\dot{\gamma }\right)$, where $\eta^{+}\left(t,\dot{\gamma }\right)$ is equivalent to zero shear viscosity at the tested shear rate. For asphalt modified by 2 wt.% B2Last, very mild stress overshoot was observed only at the highest shear rate. At a maximum used concentration of B2Last, 3 wt.%, the stress overshoot was noticeable at 2.0 s^−1^ and 5.0 s^−1^.

**Figure 11:**
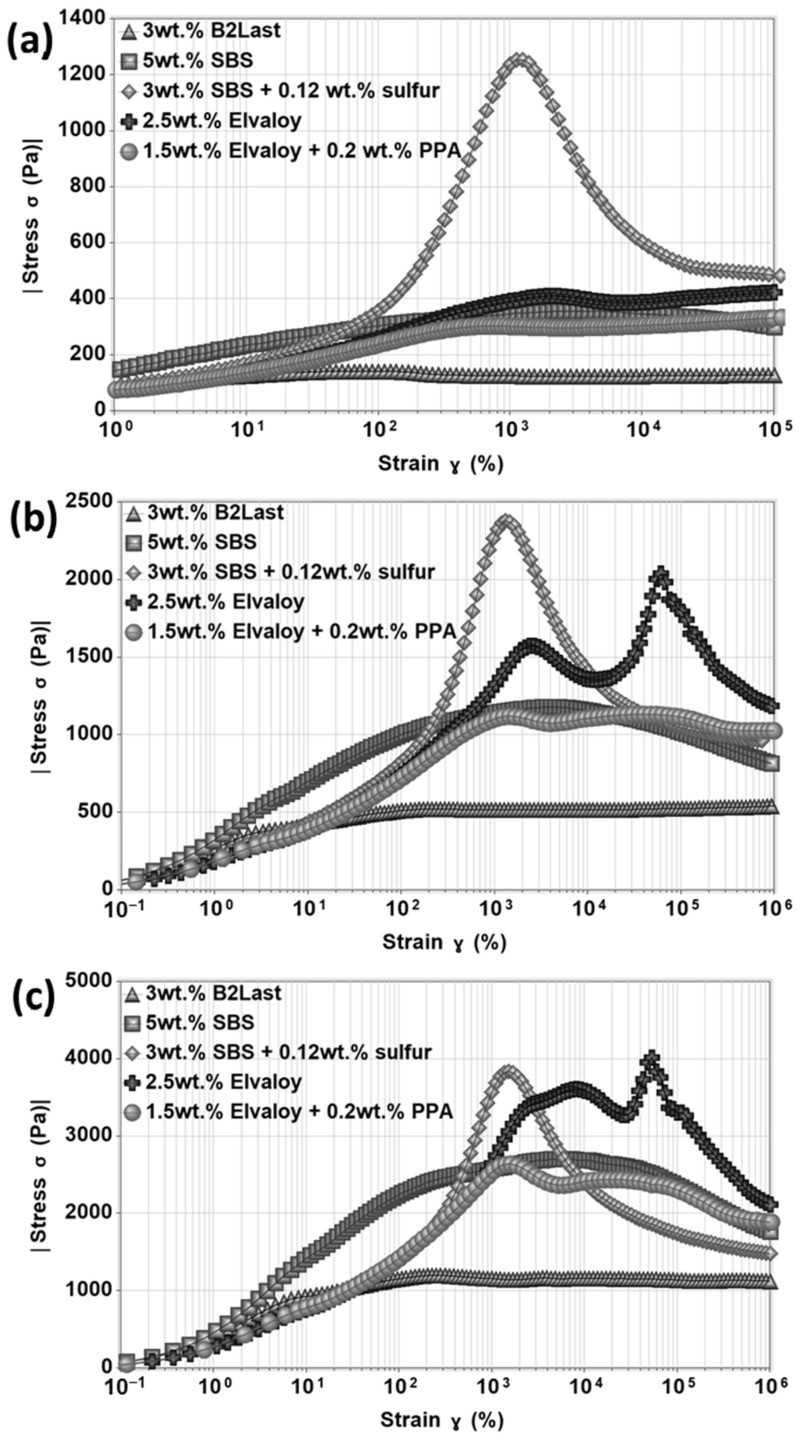
Stress overshoot as a function of strain for asphalts modified by different modification technologies, T = 60 °C: (**a**) $\dot{\gamma }=0.5\;s^{-1}$, (**b**) $\dot{\gamma }=2.0\;s^{-1}$, and (**c**) $\dot{\gamma }=5.0\;s^{-1}$.

Stress overshoot as a function of strain for asphalts modified by different modification technologies at 60 °C at three different shear rates of 0.5 s^−1^, 2.0 s^−1^, and 5.0 s^−1^ is shown in Figure 11. Similarly to differences observed in steady shear viscosity, stress growth function has shown responses strongly influenced by the type of modification. The magnitudes of $\tau^{+}$ of B2Last modified asphalts were the lowest compared to other modification technologies.

The internal network created by thermoplastic elastomer, SBS, without the presence of sulfur had a parabolic shape with stretched values of stress overshoot. This behavior could be explained by different phenomena. The swelling ability of SBS in asphalt increases the volume of the polymer phase which could lead to a reduction in asphalt’s liquid fraction. This favors aggregation and agglomeration of SBS particles [8]. Thus, the parabolic response in the start-up of the steady shear experiment can be the result of different speeds of orientation of particles of active filler in the direction of flow, breaking of large polymer droplets to smaller droplets and their subsequent deformation. The ability of polystyrene domains to split, jump, and recombine under large deformation should also be considered [25]. The formation of a three-dimensional SBS network upon vulcanization resulted in the highest magnitude of stress overshoot at all tested shear rates.

Asphalts modified by Elvaloy with and without PPA demonstrated at the start-up of steady shear experiment a peculiar stress overshoot. At a shear rate of 0.5 s^−1^, only mild stress overshoot with similar magnitude was observed for both modified asphalts. Two maxima of $\tau^{+}$ were detected when the shear rate was increased. The single stress overshoot can be generally associated with the diluted system of entanglements with high molecular weight or with a non-branched polymer system. Asphalt blend containing 2.5 wt.% of Elvaloy, however, could be in a metastable condition. The additional increase in the concentration of modifier, additional curing, or sudden change in curing conditions during preparation of modified asphalt could likely result in gelation of modified asphalt. The formed non-reversible internal network could be represented by asphaltene–Elvaloy complexes which could vary in size. Although the multiple $\tau^{+}$ overshoots could be explained by the orientation of chemically crosslinked asphaltenes and polymer complexes followed by their destruction, the origin of $\tau^{+}$ could be linked to the formation of either network or system of interacting asphaltene–Elvaloy micelles.

Due to the significantly reduced amount of Elvaloy in Elvaloy and PPA modified asphalt, the presence of multiple $\tau^{+}$ was not expected. As can be seen in Figure 11, however, this modified asphalt demonstrated two $\tau^{+}$ overshoots of the same magnitude at a shear rate of 2 s^−1^ and two $\tau^{+}$ overshoots with a larger peak of $\tau^{+}$ at 5 s^−1^. To some extent, the PPA may disintegrate already present asphaltenes and form new asphaltenes. This could help with the formation of more active asphaltene centers for reaction with oxirane present in Elvaloy and potentially more efficient curing reaction. This phenomenon could suggest a possible combination of both mechanisms of PPA in asphalt as presented by Orange et al. [46] and Baumgardner et al. [47]. The appearance of two $\tau^{+}$ overshoots in this case, however, should not be only attributed to the strength of formed chemically crosslinked asphaltene–Elvaloy complexes, but also to the increase in asphaltene micelles by PPA.

The stress growth function measured in the start-up of the steady shear experiments suggested that while asphalt modified by thermoplastic elastomer had the strongest network at all tested shear rates, the differences in the strength of the network between reactive (Elvaloy) and elastomeric modifiers seemed to be diminished at higher shear rates. Furthermore, the position of the maximum of stress overshoot in the case of reactive modifier with or without the presence of PPA was shifted to higher deformation. The appearance of the second stress overshoot also suggests an improved resistance of Elvaloy modified asphalts to deformation.

## 4. Conclusions

Two asphalt modification technologies using three modifying materials were compared. The first was a “classical” asphalt modification using SBS polyblock copolymer. The second technology used materials that react with asphalt. In one case, the material was the reactive elastomeric terpolymer Elvaloy, while in the second case, a mono- and oligomeric aromatic diisocyanate was sold under the name B2Last. In some cases, crosslinkers (with SBS) or other co-reactants (with Elvaloy) were used. Produced modified asphalts were studied and compared using the Superpave specification tests and selected rheological tests in linear and non-linear viscoelastic regions. Since B2Last as an asphalt modifier and its impact are largely unknown, this material was used in three concentrations.

### 4.1. Superpave Specification Tests

The maximum service temperature of all unaged modified asphalts increased relatively to base asphalt. After RTFOT, the maximum service temperatures of all modified asphalts containing SBS or Elvaloy (except for one) decreased. The maximum service temperature of materials modified by B2Last significantly increased.

The MSCR test has shown that crosslinked SBS and Elvaloy with PPA had low values of Jnr and high values of %Recovery. With the increasing amount of B2Last in modified asphalt, Jnr significantly improved; however, the %Recovery remained low.

According to the BBR test, the higher concentration of SBS without a crosslinker worsened the low service temperature. Elvaloy with and without PPA did not have an impact on low service temperature. In the case of B2Last with the increasing amount of modifier, the low service temperature worsened.

Viscosity at 135 °C. All studied modification systems increased values of viscosity at 135 °C, however, the effect of B2Last was negligible compared to modification technologies using SBS or Elvaloy.

### 4.2. Thermo-Rheological Testing

Material spectroscopy results suggest that the addition of B2Last creates a negligible network and rather causes an increase in the average molecular weight of modified asphalt. The test of the start-up of steady shear experiments indicates that modified asphalt with crosslinked SBS has a dense internal network, however, a low resistance to the applied rate of deformation. Elvaloy modified asphalts have a lower density of the internal network, but the network has a strong resistance to the applied rate of deformation. At higher applied shear rates, the differences in the density of internal networks between SBS and Elvaloy modifiers diminish. Results indicate an improved resistance of Elvaloy modified asphalt to deformation.

The density of the internal structure of asphalt modified by B2Last is the lowest, but it also has high resistance to higher applied shear rates.

## 5. Comments on Superpave Specification Tests

The current Superpave specification tests when used for modified asphalts have in some cases difficulties to adequately characterize modified asphalts, especially when comparing asphalts modified by different groups of modifiers. This is not only shown in comparison with thermo-rheological tests, but also in performance tests of paving mixes.

The present study points to increased stiffness in asphalt modified by reactive polymers. Our previous work showed that results from simple creep tests performed on modified asphalts correlate well with the rut resistance of paving mixes determined by the Hamburg Wheel Tester. This suggests that stiffness at high service temperatures could be more important than %Recovery_3.2kPa_.

The present method of determination of asphalt low service temperature properties does not recognize the full impact of modifiers, due to its inability to capture all material properties introduced to asphalt by modifiers (increased values of failure stress, energy and strain, and lower values of secant modulus). The DTT values were in good agreement with the low temperature values obtained on asphalt mixes with the same asphalt materials through indirect tensile testing.

As the new modification technologies are growing in popularity, the Superpave binder specification should be adapted again to adequately characterize their performance.

## Figures and Tables

**Table 1:** Properties of base asphalt (200/300 Pen grade) according to Superpave specification.

Property	Unit	Base Asphalt 200/300 Pen Grade
Original Binder		
Penetration (100 g/5 s), 25 °C	[dmm]	234
Viscosity @ 135 °C	[mPa·s]	213
Dynamic Shear $\left|G^{*}\right|/sin\delta$	[kPa]	1.00
@ Temperature	[°C]	54.6
RTFOT (Rolling Thin Film Oven Test) Residue		
Mass Loss	[%]	−0.65
Dynamic Shear $\left|G^{*}\right|/sin\delta$	[kPa]	2.20
@ Temperature	[°C]	52.9
PAV Residue		
Dynamic Shear $\left|G^{*}\right|.sin\delta$	[kPa]	5000
@ Temperature	[°C]	7.4
Creep Stiffness @ 60 s	[MPa]	278
m-Value @ 60 s		0.341
@ Temperature	[°C]	−25
Superpave Performance Grade		PG 52–34
True Superpave Grade		PG 52–35

**Table 2:** Composition of prepared polymer modified asphalt.

Polymer Modification	200/300 Pen Grade [wt.%]	Concentration of Polymer [wt.%]	Sulfur [wt.%]	PPA [wt.%]
B2Last	99	1	-	-
	98	2	-	-
	97	3	-	-
SBS	95	5	-	-
Crosslinked SBS	96.88	3	0.12	-
Elvaloy	97.5	2.5	-	-
Elvaloy with PPA	98.3	1.5	-	0.2

## Data Availability

All the data are available within the manuscript.
